# Genomic Analysis of Resistance to *Exserohilum turcicum* in Nigerien and Senegalese Sorghum Using GWAS and Machine Learning

**DOI:** 10.3390/pathogens15040389

**Published:** 2026-04-05

**Authors:** Louis K. Prom, Ezekiel J. S. Ahn, Adama R. Tukuli, Jacob R. Botkin, Sunchung Park, Lindsey C. Perkin, Clint W. Magill

**Affiliations:** 1 USDA-ARS, Southern Plains Agricultural Research Center, 2765 F & B Road, College Station, TX 77845, USA; 2 USDA-ARS Sustainable Perennial Crops Laboratory, Beltsville Agricultural Research Center, Beltsville, MD 20705, USA; 3 Department of Plant Pathology, University of Minnesota, St. Paul, MN 55108, USA; 4 Department of Plant Pathology and Microbiology, Texas A&M University, College Station, TX 77843, USA

**Keywords:** sorghum, leaf blight, *Exserohilum turcicum*, incidence, severity, Niger, Senegal

## Abstract

Sorghum, an essential crop in Niger, ranks second to pearl millet in importance for food, feed, and commerce. However, its yields are hindered by various factors, including diseases such as leaf blight caused by *Exserohilum turcicum*. In this study, field phenotypes were analyzed on 102 accessions (including checks SC748-5 and BTx623) grown and evaluated at two locations in Niger for leaf blight incidence and severity. The panel included accessions originally collected from Niger and Senegal. Genotypes were generated for 120 accessions, and GWAS/ML analyses were performed on 102 accessions due to missing phenotypic data. Among the accessions, S39, N23, and N38 exhibited mean leaf blight incidence below 50%, while S3, S43, N23, and N38 displayed the lowest severity levels, with a mean severity in Niger of 24.5 ± 0.64. Accession N23 showed relatively low incidence and severity levels across the Niger field evaluations. Using genome-wide association studies and machine learning, candidate SNPs associated with leaf blight phenotypes were identified. Genes near these SNPs were associated with functions related to plant defense mechanisms and stress responses, providing preliminary targets for future validation in sorghum leaf blight studies.

## 1. Introduction

Leaf blight on sorghum incited by *Exserohilum turcicum* (Pass.) K. J. Leonard & E. G. Suggs [syn. *Helminthosporium turcicum* (Pass.)] also occurs on maize, sudangrass, johnsongrass, teosinte, and gamagrass [1]. However, two host specificities have been described: *E*. *turcicum* f. sp. *zeae* infects maize, and the one on sorghum, *E*. *turcicum* f. sp. *sorghi* [2,3]. A single gene, SorA+ in sorghum and ZeaA+ in maize, is attributed to the host-specificity by the pathogen [3]. The sorghum leaf blight infects both young and older plants. Leaf blight symptoms ranged from large elongated to spindle-shaped spots with yellowish to gray centers with reddish margins [1,4]. Sorghum leaf blight is widely distributed in areas where sorghum is planted and under severe foliar infection, losses of up to 70% can occur [1,5,6,7]. Prom et al. [7,8] observed several prevalent diseases, including leaf blight in both Niger and Senegal. In Niger and other African countries, prevalence of leaf blight in production fields can range from 12% to 100% [5,7]. Surveys of foliar and panicle sorghum diseases during the 2019 and 2022 growing seasons in Niger and Senegal revealed 89% and 100% prevalence of leaf blight, respectively [7,8]. Meanwhile, the incidence of leaf blight among seven regions in Senegal surveyed for sorghum diseases ranged from 22% in Diourbel to 81% in Kolda [9]. Beshir et al. [5] recorded 40 to 100% leaf blight incidence on landraces planted in central Sudan. A survey conducted in Kenya by Ogolla et al. [10] documented leaf blight incidence ranging from 12% to 74% on sorghum planted in different regions.

Resistant sources of the sorghum leaf blight have been reported [5,6,11]. Hepperly and Sotomayor-Ríos [11] reported that a single dominant gene conferred resistance of SC0326 to leaf blight. Beshir et al. [12] evaluated progenies from a cross of MUC007/009 (resistant parent) × *Epuripuri* (susceptible parent) and noted that the resistance to leaf blight was quantitative.

Recently, molecular tools such as genome-wide association studies (GWAS) have been utilized to identify SNPs and locate genes associated with economically important traits such as disease resistance. In addition to evaluating the Nigerien and Senegalese sorghum accessions for incidence and severity of leaf blight in two locations in Niger (Bengou and Maradi), this work also reports the results of GWAS and a CatBoost-based machine learning (ML) approach to identify candidate SNP markers associated with leaf blight incidence and severity. CatBoost was selected due to its robustness in high-dimensional settings, built-in regularization, and ability to efficiently handle mixed feature representations without extensive preprocessing [13].

## 2. Materials and Methods

Study area: The research fields were established in Niger, West Africa, during the 2022 growing season. Niger lies at 17°35′48.77″ N latitude and 8°04′58.26″ E longitude [14]. In Niger, accessions were planted in two locations: (1) Bengou, in the Southern Dosso region 13°02′56.40″ N and 03°11′37.32″ E with a Sahelian and a Sahelo-soudanian climate, and (2) Maradi 13°30′0.00″ N and 07°06′06.26″ E, in Maradi region, with a Sahelian climate [15]. The soil type in the Maradi region is ferruginous, while the soil type in the Dosso region is hydromorphic [16]. A total of 102 accessions from Niger and Senegal, including checks SC748-5 and BTx623, were planted at each location. Seeds of each accession were planted in 1.8 m rows with 0.8 m row spacing at each location. Accessions were planted in a randomized complete block design, and each accession was replicated three times. Fields were kept free of weeds with occasional hand hoeing. Plants were evaluated for leaf blight incidence in the soft to early hard dough stage of development. The leaf blight incidence was based on the formula noted below.

$$\mathrm{Incidence}=\frac{\mathrm{Number}\;\mathrm{of}\;\mathrm{plants}\;\mathrm{with}\;\mathrm{the}\;\mathrm{disease}\;\mathrm{in}\;a\;\mathrm{row}}{\mathrm{Number}\;\mathrm{of}\;\mathrm{plants}\;\mathrm{assessed}\;\mathrm{in}\;a\;\mathrm{row}.}\;\times 100$$

Disease Severity Scale: The severity scale was previously described by Prom et al. [7,17] and based on 0–11 with mid-points, where 1 = 5.5, 2 = 15.5, 3 = 25.5, 4 = 35.5, 5 = 45.5, 6 = 55.5, 7 = 65.5, 8 = 75.5, 9 = 85.5, 10 = 95.5, and 11 = 100 used to calculate the mean severity.

### 2.1. GWAS

DNA extraction for 120 sorghum accessions (60 Niger, 60 Senegal) was performed; downstream association and ML analyses used the subset with matched field phenotypes (n = 102). In brief, it was performed using either NucleoSpin Plant II kits (Macherey-Nagel, Düren, Germany, ref. 740770) or a modified CTAB protocol following the established methods of Prom et al. [17], Kale et al. [18], and Doyle and Doyle [19]. The DNA was purified using 7.5 M ammonium acetate and isopropanol. OD ratios (260/230, 260/280) and examined with SpectraMax^®^ QuickDrop™ Micro Volume spectrophotometer (Molecular Devices, San Jose, CA, USA). Then, the samples were run on a 1% agarose gel stained with ethidium bromide for quality control. The sequencing was performed at Texas A&M (TxGen, 1500 Research Pkwy Suite 250, College Station, TX, USA) using Illumina NovaSeq 6000 (2.6× average coverage). Before library preparation, the samples were repurified. The sequencing raw data was processed using the GATK (Genome Analysis Tool Kit, Broad Institute, Cambridge, MA, USA) best practices for variant calling implemented in the DRAGEN platform (Illumina, San Diego, CA, USA). The sequences were then aligned against *Sorghum bicolor* v3.1.1 available at Phytozome (https://phytozome-next.jgi.doe.gov/info/Sbicolor_v3_1_1, accessed on 1 May 2023) as the reference genome for SNP calling [20]. The calls were filtered using the following parameters: Minimum coverage depth = 3 and minimum genotype quality = 9 on a Phred scale. The resulting variants were then filtered to only accept SNPs with a minimum minor allele frequency of 0.05 and a maximum rate of missing data of 0.5. Variant filtration was conducted by using bcftools. The SNP data were finally imputed using Beagle (Beagle 8.7e1.jar) [21]. PLINK v1.9 [22] was used for VCF file conversion, and for computational efficiency we randomly subsampled 500,000 SNPs from the full genotypic dataset for downstream GWAS and ML analyses.

**Figure 1:**
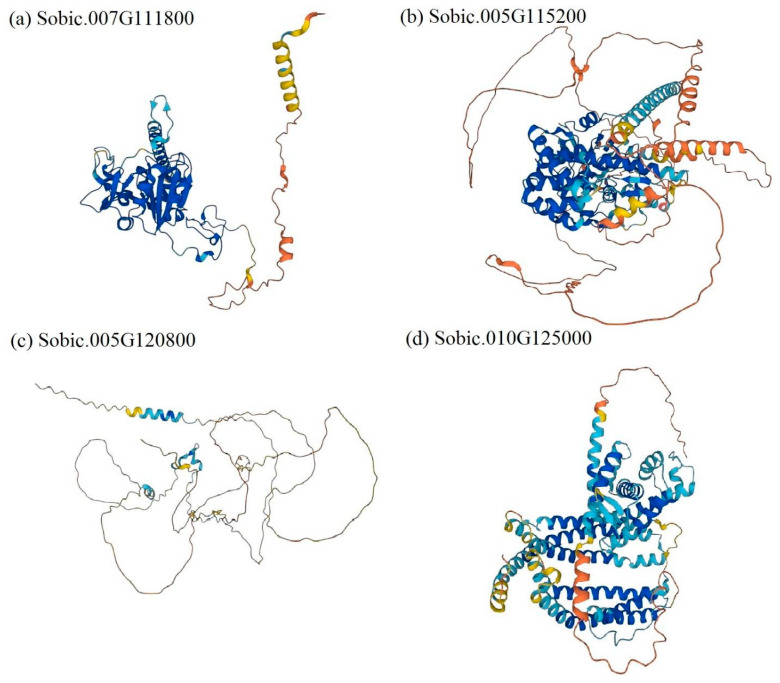
Predicted protein structures of top candidate genes. (**a**) *Sobic.007G111800*, (**b**) *Sobic.005G115200*, (**c**) *Sobic.005G120800*, and (**d**) *Sobic.010G125000*. Model confidence—blue: very high (pLDDT > 90), sky blue: confident (90 > pLDDT > 70), yellow: low (70 > pLDDT > 50), and orange: very low (pLDDT < 50). pLDDT: per-residue confidence score between 0 and 100. The predicted protein structure of *Sobic.007G116000* is unavailable.

For GWAS, GEMMA v0.98.3 [23] was used by conducting a univariate linear mixed model association testing, accounting for the relatedness matrix as a covariate term and employing the Wald test for determining statistical significance. The software package vcf2gwas v0.8.3 generated phenotype and genotype distribution [24]. Top candidate SNPs from the GWAS were tracked by searching the reference sorghum genome sequence version 3.1.1, accessed through the JGI Phytozome 13 website. Available predicted protein structures of top candidate genes identified by GWAS were retrieved from the AlphaFold Protein Structure Database (https://alphafold.ebi.ac.uk/ accessed on 8 May 2023) [25] using the closest available Arabidopsis orthologs, and were used for qualitative structural context (Figure 1). Available predicted protein network nodes of top candidate genes were searched through the STRING database version 11.5 website (https://string-db.org/ accessed on 5 May 2023) [26].

### 2.2. Data Merging, Preprocessing, and Standardization for ML

The analysis integrated genotype and phenotype data to create a unified dataset. SNP marker positions, extracted from the VCF file, were transposed so that individual samples (cultivars) were represented as rows and SNP markers as columns. This transposed genotype matrix was merged with phenotype data based on shared sample identifiers. Phenotype data included two traits: LB-Incidence (incidence of leaf blight) and LB-Severity (severity of leaf blight). Each trait was analyzed independently by defining it as the target variable while using SNP markers as features. Genotype data were encoded as allele dosages (0, 1, 2), representing the number of alternative alleles present at each SNP locus (homozygous reference = 0, heterozygous = 1, homozygous alternative = 2). This encoding aligns with the additive genetic framework in quantitative genetics, where allele dosage is assumed to contribute proportionally to phenotypic variation. While these values are numerical, they represent discrete genotype states rather than continuous measurements, allowing models such as CatBoost to effectively capture non-linear relationships without requiring extensive feature transformation. Prior to model training, the target variables were standardized to have zero mean and unit variance using StandardScaler from the sklearn library [27]. To avoid data leakage, scaling parameters were learned exclusively from the training folds and applied to the corresponding validation folds within each cross-validation iteration.

### 2.3. ML Model Training, Evaluation, and Feature Importance Analysis

CatBoost [13] was selected due to its strong performance on high-dimensional datasets, its ability to model complex non-linear relationships, and its robustness in handling structured data without extensive preprocessing. These characteristics make it particularly suitable for genomic datasets, where the number of features (SNP markers) greatly exceeds the number of samples. Separate models were trained for LB-Incidence and LB-Severity using SNP markers as predictive features. The CatBoostRegressor was implemented using default hyperparameters, with verbose = 50 and thread_count = −1 specified to enable training monitoring and efficient CPU utilization. Accordingly, parameters such as learning rate, tree depth, number of iterations, and regularization strength were automatically determined by the algorithm and were not manually tuned. Default hyperparameters were intentionally used to ensure reproducibility and consistency across both traits and to reduce the risk of overfitting associated with extensive hyperparameter optimization in high-dimensional datasets with relatively small sample sizes. Given that the primary objective of this analysis was feature ranking and candidate SNP prioritization rather than predictive optimization, this approach provides a stable and interpretable baseline.

Model performance was evaluated using 10-fold cross-validation. Root Mean Squared Error (RMSE) was calculated for each fold based on predictions for held-out data, and the mean RMSE across folds was reported as the overall performance metric. Feature (genomic position) importance scores were computed using the trained CatBoost models to identify SNP markers most relevant to each trait. Importance scores were normalized to facilitate comparison across features. Cumulative importance was then calculated to determine the minimum number of SNP markers required to explain 80% of the total importance. These features were extracted and visualized using cumulative importance plots. For each trait, the top 20 SNP markers were identified and visualized using bar plots to highlight the most predictive genomic positions. While no overlap was observed among the top 20 SNPs between LB-Incidence and LB-Severity, extending the analysis to SNPs within the 80% cumulative importance threshold revealed shared markers between traits. This broader ranking approach reflects the quantitative and polygenic nature of leaf blight resistance and enables flexible prioritization of candidate SNPs for downstream validation.

## 3. Results

### 3.1. Leaf Blight Incidence and Severity in Nigerien Sorghum Germplasm

Leaf blight incidence and severity were evaluated across two locations in Niger (Bengou and Maradi) (Table 1 and Table 2). The mean incidence was 81.26 ± 1.37. Among the accessions evaluated, only S39, N23, and N38 had mean leaf blight incidence below 50%. Overall, the mean severity level in Niger was 24.5 ± 0.64. Accessions S3, S43, N23, and N38 exhibited the lowest mean severity when tested against the leaf blight-causing pathogen *E*. *turcicum*.

**Figure 2:**
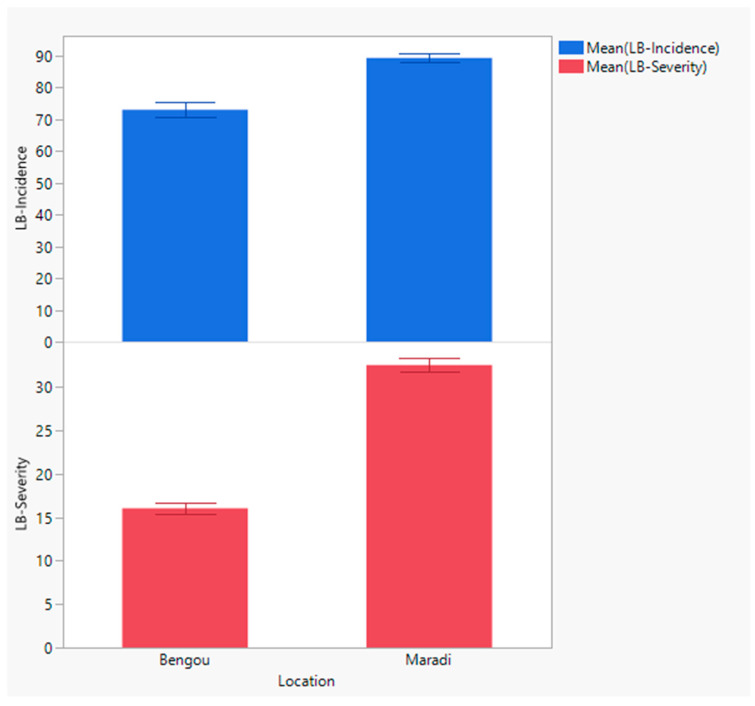
Leaf blight incidence (blue) and severity (red) differed between the two Niger locations (Bengou and Maradi) based on plot-level observations (two-sample *t*-test, *p* < 0.0001).

Mean leaf blight incidence and severity were compared between Bengou and Maradi (Figure 2), using plot-level observations grouped by location. Both traits were significantly lower in Bengou (incidence: 72.94 ± 1.91; severity: 16.04 ± 0.76) than in Maradi (incidence: 89.17 ± 1.86; severity: 32.54 ± 0.76; two-sample *t*-test, *p* < 0.0001), indicating a significant location effect.

**Figure 3:**
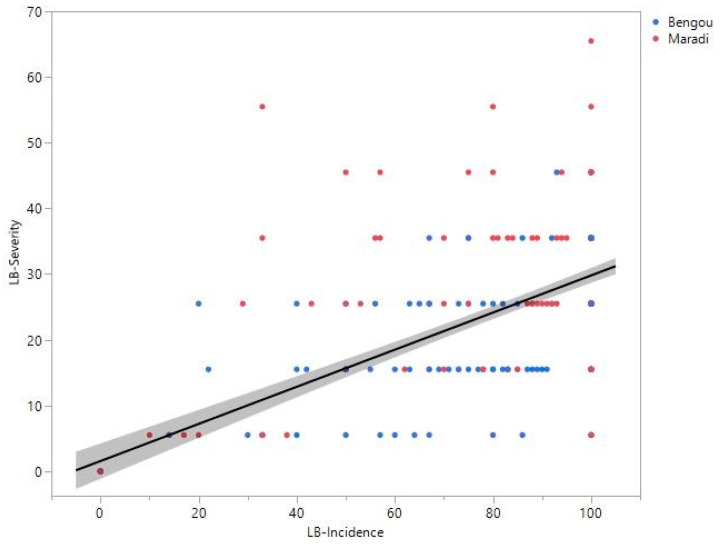
Pearson’s correlation between accession-level mean incidence and mean severity in Niger was *r* = 0.61 (*p* < 0.0001). Points are colored by location.

Pearson’s correlation showed a positive association between accession-level mean incidence and mean severity (*r* = 0.61, *p* < 0.0001; Figure 3), indicating that accessions with higher incidence tended to exhibit higher severity.

### 3.2. GWAS of Leaf Blight Incidence

**Figure 4:**
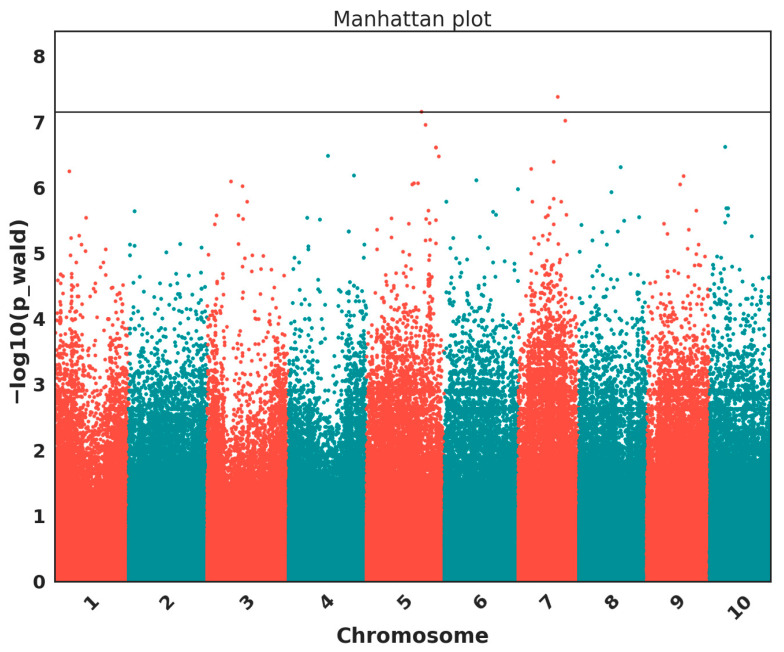
Genome-wide association results for leaf blight incidence in Niger. The Manhattan plot shows two SNPs exceeding the Bonferroni threshold on chromosomes 7 and 5.

**Figure 5:**
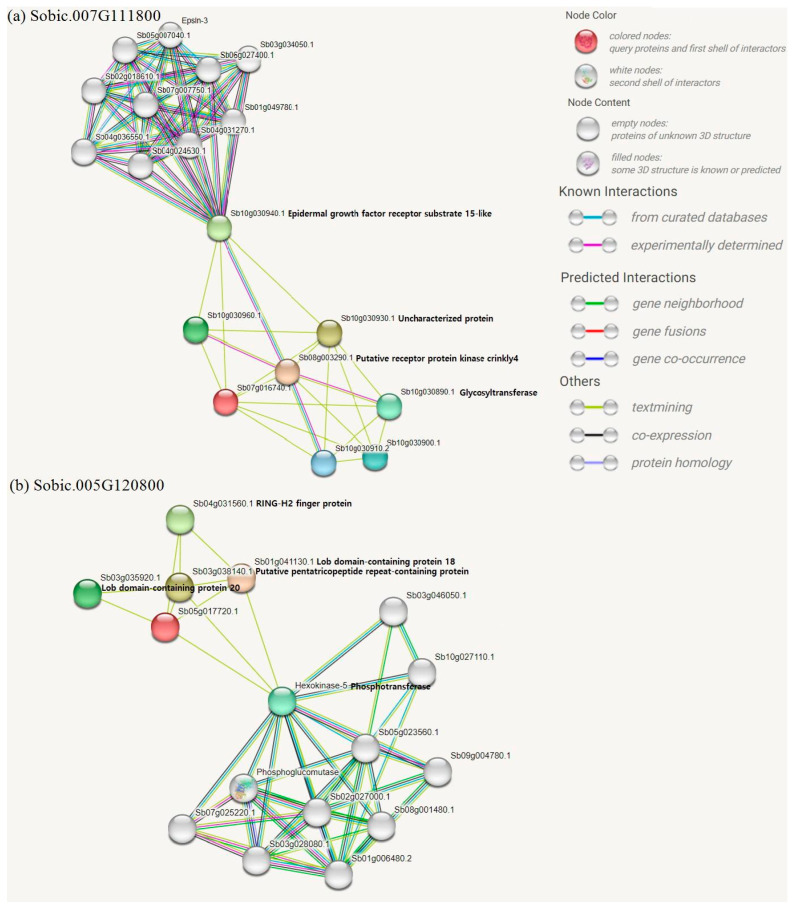
Predicted protein network nodes of top candidate genes. (**a**) *Sobic.007G111800* and (**b**) *Sobic.005G120800*. Red beads indicate the candidate genes.

GWAS analysis identified two SNPs passing the Bonferroni threshold (S07_42352720 and S05_47989804) (Figure 4 and Table 3). Figure 4 displays the Manhattan plot, and Table 3 lists the nearby annotated genes for the top SNPs identified in this study. Figure 1 illustrates the protein structures of the top candidate genes generated based on Arabidopsis orthologs through the AlphaFold Protein Structure Database. Figure 5 shows predicted protein–protein interaction networks associated with selected candidate genes for leaf blight incidence in Niger. As not all candidate genes were available in AlphaFold and STRING databases, Figure 1 and Figure 5 show the data for a few candidate genes.

### 3.3. Identification of Predictive SNP Markers for Leaf Blight Resistance Using ML

**Figure 6:**
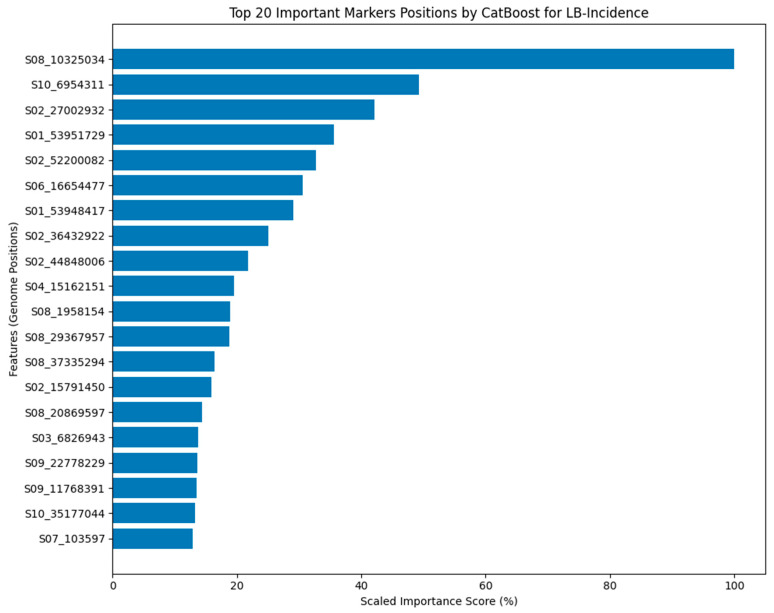
Top 20 SNP Markers Associated with Leaf Blight Incidence in Sorghum as Determined by CatBoost Algorithm. Importance scores of the top 20 SNP markers significantly associated with LB incidence in Nigerien and Senegalese sorghum accessions. Marker names indicate chromosome number and base pair position (e.g., S08_10325034 refers to chromosome 8, position 10,325,034).

To further examine genomic predictors associated with leaf blight incidence and severity, we employed the CatBoost ML algorithm to rank SNP markers for both traits. Given the high-dimensional nature of the SNP dataset relative to the number of accessions, this analysis was designed to prioritize informative markers based on their relative contribution to phenotypic variation rather than to optimize predictive performance. Model performance was evaluated using 10-fold cross-validation, with Root Mean Squared Error (RMSE) calculated on held-out folds and averaged across iterations. The CatBoost models yielded mean RMSE values of 0.74 for LB-Incidence (Supplementary Figure S1a) and 0.94 for LB-Severity (Supplementary Figure S1b), indicating moderate predictive performance consistent with the quantitative and polygenic nature of these traits. An 80% cumulative importance threshold was used to identify the most influential SNP markers contributing to LB-Incidence (Supplementary Figure S1c; Supplementary Table S1) and LB-Severity (Supplementary Figure S1d; Supplementary Table S2). This threshold summarizes the subset of markers accounting for the majority of the feature-importance mass in the CatBoost models and provides a biologically meaningful set of candidate loci for downstream investigation. The CatBoost model prioritized a set of SNP markers for leaf blight incidence (Figure 6). The top 20 markers, ranked by their scaled importance scores, are distributed across multiple chromosomes, supporting a complex and potentially polygenic genetic architecture underlying this trait. Notably, marker S08_10325034 exhibited the highest importance score, accounting for approximately 100% of the scaled importance, followed by S10_6954311 and S02_27002932, with importance scores of approximately 49.2% and 42.1%, respectively. The remaining markers in the top 20 displayed importance scores ranging from 35.6% to 12.9% (Figure 6).

**Figure 7:**
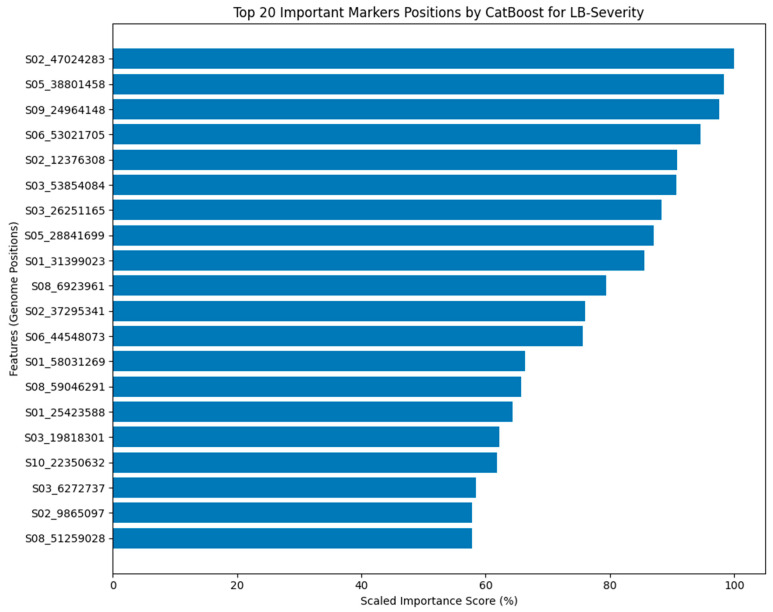
Top 20 SNP Markers Associated with Leaf Blight Severity in Sorghum as Determined by CatBoost Algorithm. The top 20 most important SNP markers for predicting LB severity in a panel of Nigerien and Senegalese sorghum accessions, as determined by the CatBoost algorithm. The scaled importance score (%) represents the relative contribution of each marker to the model’s predictive accuracy. Marker labels indicate chromosome and position (e.g., S05_38801458 represents chromosome 5, position 38,801,458).

To identify potential candidate genes associated with these predictive markers, we examined the genomic regions surrounding the top five SNPs for LB-Incidence (Table 4). These regions harbor genes with diverse predicted functions, including a serine carboxypeptidase, a flavonol-3-O-glycoside-7-O-glucosyltransferase, an LETM1-like protein, an oligopeptide transporter, and a phosphatidate cytidylyltransferase. Similarly, the CatBoost analysis for leaf blight severity revealed a distinct set of prioritized SNP markers (Figure 7). The top 20 markers for severity also spanned multiple chromosomes and exhibited a range of importance scores, further supporting a complex genetic basis. Marker S02_47024283 had the highest importance score of approximately 100%, followed by S05_38801458 and S09_24964148, with importance scores of approximately 98.4% and 97.5%, respectively. The importance scores for the remaining top 20 markers ranged from 94.6% to 57.8% (Figure 7). The top five SNPs associated with LB-Severity were also linked to nearby candidate genes (Table 4). These include genes encoding an embryo-defective protein, an uncharacterized protein, a cis-zeatin O-beta-D-glucosyltransferase, and a leucine-rich repeat (LRR) protein. A comparison of the top 20 marker sets for incidence and severity revealed no overlap between the two traits, suggesting that these phenotypes may capture partly distinct genomic signals in this dataset. However, two SNPs—S02_50596409 (associated with nearby genes including members of the DVL family and glutathione S-transferase) and S02_54879183 (near a gene encoding a glucosyltransferase)—exhibited moderately high importance scores in both the incidence and severity models, despite not being among the top 20 markers for either trait. This observation suggests that while the primary genetic factors influencing incidence and severity may be largely distinct, a subset of genomic regions may contribute to both traits at moderate effect sizes. Overall, these results highlight the utility of the CatBoost framework for prioritizing candidate SNPs in complex, quantitative disease resistance traits, while emphasizing that the identified markers represent ranked candidates requiring further validation rather than definitive causal loci.

## 4. Discussion

The impact of climate change, coupled with population growth to around 9.1 billion by 2050, will require increases in crop production, including cereals such as sorghum for food, feed, and other uses [28]. Increasing sorghum production in regions affected by fungal diseases, including *E. turcicum*, will require integrated disease management strategies, including the identification of resistant germplasm [1,8,29]. Sorghum plays an integral role in the lives of millions of inhabitants in Niger and is used primarily for food, feed, and commerce [30,31,32]. The crop ranks behind pearl millet in importance [30,31]. However, sorghum yields in Niger are still low due to several factors, including diseases such as leaf blight [7]. During the 2019 and 2022 surveys of sorghum diseases across major production regions in Niger, the prevalence of leaf blight was 89% and 100%, respectively [7,8]. This disease is widespread in all sorghum growing regions of Niger, suggesting that evaluating accessions for resistance under natural infection or inoculation with the pathogen can both be effective. However, differences in leaf blight incidence and severity between Bengou, Dosso region and Maradi, Maradi region were noted. These differences could be attributed to the differences in weather patterns as recorded in Table 5 [8] or the pathotypes that exist in the two locations. This study’s mean leaf blight incidence rate was 81.26 ± 1.37, while the mean severity level in Niger was 24.5 ± 0.64. These values can be viewed alongside the previously reported Senegal evaluation of the same accessions [17], although the present study focuses on field performance observed in Niger. The mean incidence of leaf blight was 87% across five regions in Niger surveyed for sorghum diseases in 2022, while the mean severity was 22% [7]. Some regions have reported leaf blight-resistant sorghums, including Puerto Rico, Sudan, and Ethiopia [5,6,11]. Among the accessions evaluated in Senegal and Niger, N30 from Niger exhibited low leaf blight severity in Senegal [17]. GWAS is a promising approach to genetic analysis and has proven to be a valuable tool in identifying candidate genes for many plant traits [33]. The same accessions used in this study were planted in three locations in Senegal, and the GWAS identified six SNPs associated with the average leaf blight incidence rate [17]. In the Senegal GWAS, the candidate genes were found in chromosomes 2, 3, 5, 8, and 9 [17], while the SNPs in the current Niger study were found in chromosomes 5, 7, and 10. Using three tropical maize germplasm mapping panels evaluated against *Setosphaeria turcica* causal agent of Northern corn leaf blight (NCLB), a GWAS identified 22 SNPs significantly associated with NCLB response [34], while Ding et al. [35] noted 12 and 10 loci that were significantly associated with NCBL resistance. Candidate genes found in both sorghum and maize in response to leaf blight are reported to play a role in resistance to both crops [36]. Lipps et al. [37] evaluated two sorghum recombinant inbred line populations for response against *E. turcicum* and detected six QTLs. All these GWAS on sorghum and maize against *E. turcicum* will continue to enhance our knowledge of the genes involved in resistance mechanisms in these two economically important crops. Because incidence and severity differed significantly between Bengou and Maradi, environmental heterogeneity likely contributed to phenotypic variation in this study. Accordingly, the present GWAS based on accession-level means across locations should not be interpreted as a formal genotype-by-environment analysis.

Herein, GWAS identified candidate SNPs associated with leaf blight incidence in Niger. The closest annotated gene from S07_42352720 is lysine ketoglutarate reductase trans-splicing related 1 (DUF707). In a transcriptome study of rice (*Oryza sativa*), DUF707 was highly expressed when exposed to herbicide [38]. Moreover, as displayed in Figure 5a, the predicted protein network nodes of the gene include protein kinase crinkly4 and glycosyltransferase. Glycosyltransferases have been reported to be top defense-related genes against fungal pathogens in sorghum and have an essential role in plant defense and stress tolerance [39,40]. The second listed candidate gene is *Sobic.005G115200*, which contains the ring finger domain. RING finger proteins are essential in governing growth and development, hormone signaling, and controlling responses to biotic and abiotic stresses in plants [41]. The third listed gene is zinc finger containing *Sobic.007G116000*. These plants also showed constitutive up-regulation of multiple defense-related genes. In *Nicotiana benthamiana*, zinc-finger protein plays a crucial role in activating the pathogen defense response in plants [42]. Phosphofructokinase was the closest gene to the SNP locus S05_53064984. Phosphofructokinase is critical in sugar metabolism and is closely linked to drought stress responses in cotton (*Gossypium arboreum* L.) [43]. Predicted protein network nodes of the gene were also highly linked with ring-h2 finger protein, lateral organ boundaries (LOB) domains, and hexokinase 5. LBD proteins have been well documented, with biological roles in plant development and defense response processes [44]. Likewise, mitochondria-associated hexokinases control programmed cell death in *N. benthamiana* [45]. Plant steroids (*Sobic.010G125000*) are perceived by cell surface receptors that contain transmembrane receptor serine/threonine kinases that play a central role in signaling during pathogen recognition, the subsequent activation of plant defense mechanisms and developmental control [46,47,48].

While GWAS has been a valuable tool for identifying candidate genes associated with important plant traits [33], this study employed both GWAS and a machine learning (CatBoost) approach to identify SNP markers associated with leaf blight resistance in Nigerien and Senegalese sorghum germplasms. Complementing the GWAS results, the CatBoost analysis identified a distinct set of SNP markers with high predictive importance for both leaf blight incidence and severity (Figure 6 and Figure 7). Notably, there was no overlap between the significant SNPs identified by traditional GWAS and the top-ranked markers identified by the CatBoost algorithm for either incidence or severity. This lack of concordance between the two analytical approaches suggests that ML methods such as CatBoost may prioritize genomic regions not highlighted by conventional GWAS in this dataset, potentially due to their ability to capture non-linear effects and interactions among loci. This pattern further indicates that incidence and severity may require separate consideration in future marker prioritization and validation efforts. It is important to note that the CatBoost framework in this study was applied primarily as a feature-ranking tool rather than a fully optimized predictive model. Given the high dimensionality of the SNP dataset relative to the number of accessions, and the quantitative nature of the traits, the analysis focused on identifying and prioritizing candidate SNPs based on their relative contribution to phenotypic variation. Although the models demonstrated moderate predictive performance (as reflected by cross-validated RMSE values), these results are consistent with expectations for complex polygenic traits influenced by many loci with small to moderate effects. Therefore, the identified SNPs should be interpreted as prioritized candidates rather than definitive causal variants.

For leaf blight incidence, the CatBoost model highlighted a region on chromosome 8 containing a gene encoding a serine carboxypeptidase 1 precursor (*Sobic.008G073700*) near the most predictive SNP marker (S08_10325034, Table 4). Serine carboxypeptidases and their close relatives, serine carboxypeptidase-like proteins, are known to play multifaceted roles in plant growth, development, and stress responses [49]. A comprehensive study of the SCPL gene family in soybean identified 73 SCPL genes and demonstrated their involvement in resistance to both biotic and abiotic stresses, including nematode infection, drought, salinity, and cold [49]. Another notable candidate gene identified for incidence is *Sobic.010G081600*, encoding a flavonol-3-O-glycoside-7-O-glucosyltransferase (Table 4). Flavonoids are a diverse group of secondary metabolites known to play significant roles in plant defense against both biotic and abiotic stresses [50]. The role of flavonol-3-O-glycoside-7-O-glucosyltransferase in modifying flavonoids suggests that it may influence the accumulation or activity of specific flavonoid compounds contributing to leaf blight resistance in sorghum [50].

For leaf blight severity, a gene encoding a leucine-rich repeat (LRR) protein (*Sobic.002G104500*) was identified near a highly ranked SNP marker for severity (S02_12376308, Table 4). LRR proteins, particularly those belonging to the LRR receptor-like kinase (LRR-RLK) family, are known to function as pattern recognition receptors (PRRs) in plants, playing crucial roles in the perception of pathogen-associated molecular patterns (PAMPs) and the activation of downstream defense responses [51]. The identification of an LRR protein gene near a highly ranked SNP marker suggests that this protein may be involved in the recognition of *Exserohilum turcicum*-derived PAMPs and the initiation of defense signaling pathways in sorghum [51].

In this study, top candidate genes identified through GWAS and CatBoost were associated with functions related to plant defense mechanisms and stress responses, making these accessions valuable candidates for follow-up evaluation in sorghum leaf blight research. The use of CatBoost, in particular, highlighted additional markers and nearby candidate genes not prioritized by GWAS alone, supporting the value of complementary analytical approaches for leaf blight-associated traits. Additionally, it is important to consider other genes identified through the STRING database that are connected to the top candidates. Functional validation remains essential to confirm the biological relevance of these associations. However, gene editing in sorghum using CRISPR/Cas9 remains technically challenging due to its monocot nature. Expanding the number of sorghum accessions from Niger and Senegal and identifying overlapping signals across independent studies will be critical for refining candidate gene selection, while advances in gene-editing technologies may facilitate future functional validation.

GWAS has been instrumental in identifying genetic variations linked to specific phenotypes, such as candidate genes for blackleg resistance in *Brassica juncea* [52]. However, GWAS is limited in detecting rare genetic variants and often struggles to capture non-linear effects of genomic variation on traits [53]. While GWAS has identified numerous trait-associated loci, the causal genes within these loci often remain unclear, making functional validation challenging [54]. In contrast, machine learning offers a complementary framework by leveraging high-dimensional genomic data to uncover complex genotype–phenotype relationships [55]. ML approaches can capture subtle genomic signals, including non-linear interactions and small-effect variants, thereby enhancing our understanding of complex traits such as disease resistance [56]. By integrating genomic data, ML models can prioritize genes and alleles contributing to defense mechanisms, offering an alternative perspective to traditional association methods. While ML has been widely applied to agronomic traits such as yield and flowering time, its application to disease resistance remains relatively limited. Emerging studies in crops such as rice, wheat, maize, and sugarcane highlight its potential, although no single method is universally optimal, emphasizing the need for context-specific and integrative analytical strategies [57].

For leaf blight resistance in sorghum, the differences in SNP positions identified by GWAS and CatBoost highlight these methodological contrasts. GWAS relies on single-marker linear regression and may overlook loci with polygenic effects or small contributions. In contrast, CatBoost employs ensemble learning and non-linear modeling, enabling the detection and prioritization of SNPs associated with both LB-Incidence and LB-Severity. The inability of GWAS to identify Bonferroni-significant markers for LB-Severity, together with the CatBoost prioritization of markers for both traits, suggests that the two approaches capture different aspects of the genetic architecture. These findings support the interpretation that GWAS and ML methods are complementary rather than competing approaches for dissecting complex disease resistance traits.

## 5. Conclusions

This study evaluated 102 sorghum accessions from Niger and Senegal (including checks) for leaf blight incidence and severity across two field locations in Niger, revealing substantial phenotypic variation for both traits. Several accessions, including S39, N23, and N38, exhibited strong resistance, with mean leaf blight incidence below 50%, while S3, S43, N23, and N38 showed the lowest severity levels. These accessions represent valuable genetic resources for sorghum improvement programs targeting disease resistance. By integrating traditional GWAS with a machine learning (CatBoost) framework, this study identified complementary sets of candidate SNP markers and nearby genes associated with leaf blight incidence and severity. Notably, the lack of overlap between the top-ranked markers for incidence and severity, as well as between GWAS and CatBoost results, suggests that these traits may be governed by partly distinct and complex genetic architectures. The CatBoost analysis further prioritized additional genomic regions not captured by GWAS alone, highlighting the value of ML approaches in uncovering non-linear and polygenic signals in high-dimensional genomic data. Importantly, the ML framework was applied as a feature-ranking and candidate prioritization tool rather than a fully optimized predictive model. As such, the identified SNPs should be interpreted as prioritized candidates for further investigation rather than confirmed causal variants. The integration of GWAS and ML thus provides a complementary and scalable strategy for identifying genomic regions associated with complex disease resistance traits. Overall, these findings contribute to a better understanding of the genetic basis of leaf blight resistance in sorghum and provide a set of candidate markers for future research. However, validation in larger and independent populations, as well as functional characterization of candidate genes, will be essential before these markers can be effectively deployed in breeding programs.

## Figures and Tables

**Table 1:** The average leaf blight incidence rate across the two locations in Niger was ordered from high to low (the overall mean is shown in the final row for reference). The standard error of the mean is listed next to the average value.

Accessions	Incidence	SEM	Accessions	Incidence	SEM
N4	100.00	0.00	S29	81.00	10.21
N29	100.00	0.00	S40	80.60	13.24
N46	100.00	0.00	S9	80.17	16.09
S2	100.00	0.00	S4	80.00	20.00
S6	100.00	0.00	S14	80.00	16.13
S36	100.00	0.00	S35	80.00	20.00
S42	100.00	0.00	S45	80.00	16.33
S49	100.00	0.00	S48	80.00	16.33
S65	100.00	0.00	S56	80.00	13.66
N6	98.00	2.00	S15	79.57	14.58
S37	97.80	2.20	S10	78.80	16.66
BTx623	97.60	2.40	S22	78.50	16.40
N43	97.20	2.80	N19	78.17	15.95
S58	97.20	2.80	N36	78.17	16.43
S38	96.00	4.00	N50	77.83	16.47
N25	95.60	4.40	N27	77.50	15.90
S32	95.25	4.75	S27	77.50	15.90
S19	95.00	5.00	S13	77.00	13.33
S33	95.00	5.00	S57	76.71	9.65
S34	95.00	3.52	S55	76.33	15.91
S59	95.00	5.00	SC748-5	76.20	10.56
S7	94.83	5.17	N40	76.17	9.78
S31	94.50	5.50	N53	75.83	16.20
S44	94.00	6.00	N26	75.80	16.87
S17	92.86	7.14	N30	75.00	25.00
N18	92.17	7.83	N41	75.00	17.08
N60	91.83	4.13	S1	75.00	17.08
N3	90.67	5.91	S5	75.00	19.36
N20	90.33	7.24	S50	75.00	25.00
N9	90.00	6.63	N28	74.20	18.85
N42	90.00	10.00	S23	73.83	16.77
S8	90.00	6.07	S60	72.57	15.31
S28	90.00	6.83	N5	71.40	14.52
S47	90.00	10.00	N2	71.00	19.01
S30	89.33	5.96	S12	70.17	18.04
N48	88.60	7.87	S54	69.00	19.69
N45	88.33	9.80	N54	68.83	17.38
S51	88.00	12.00	S46	68.50	23.21
S11	87.50	7.92	S18	66.67	21.08
S52	87.25	8.73	N58	64.83	20.58
S41	86.33	8.27	N24	64.00	20.40
N44	86.00	8.07	N51	63.83	20.36
N55	85.83	9.81	N52	60.00	24.49
N39	84.60	11.63	N22	56.80	20.79
N57	83.67	8.74	S3	53.40	22.62
N8	83.60	7.55	S16	50.00	22.36
N49	83.33	16.67	S43	50.00	22.36
N56	83.33	16.67	S39	49.17	18.37
N59	83.33	10.54	N23	48.00	18.52
S21	83.33	16.67	N38	25.00	25.00
S20	81.33	11.91	Average	81.26	1.37
N34	81.17	8.52			

**Table 2:** The average leaf blight severity level across the two locations in Niger was ordered from high to low (the overall mean is shown in the final row for reference). The standard error of the mean is listed next to the average value.

Accessions	Severity	SEM	Accessions	Severity	SEM
S37	39.50	5.10	S12	24.58	7.41
S49	38.83	8.82	N2	24.40	9.07
S33	38.00	4.79	S50	24.13	8.38
S38	37.50	7.35	N6	23.83	4.77
N34	37.17	6.01	S36	23.50	2.00
S32	35.50	10.80	S44	23.50	5.83
S30	33.83	4.77	S52	23.00	6.29
N42	33.50	3.74	N19	22.92	9.15
N43	33.50	7.35	N53	22.92	6.61
S4	33.00	7.50	N56	22.92	7.55
S6	32.17	6.67	S1	22.92	5.51
S42	32.17	6.15	N28	22.40	9.23
S46	31.63	12.25	N40	22.17	2.11
BTx623	31.50	2.45	N60	22.17	2.11
S2	31.50	10.30	N51	22.00	8.01
S34	31.50	4.00	N26	21.50	6.78
S58	31.50	10.30	S51	21.50	5.10
N18	30.50	5.63	SC748-5	21.50	5.10
N50	29.58	6.95	S16	21.30	9.25
N4	29.50	2.45	N41	21.25	8.45
N46	29.50	6.78	S14	21.25	6.17
S10	29.50	8.12	S22	21.25	4.97
S47	29.50	8.12	N45	20.50	5.00
S60	29.00	6.30	S11	20.50	5.00
N3	28.83	3.33	S65	20.50	5.00
N44	28.83	7.15	S27	19.58	7.62
S21	28.83	12.02	S48	19.58	6.68
S15	27.57	7.90	N9	19.50	5.10
N8	27.50	8.00	S40	19.50	5.10
N48	27.50	4.90	N52	19.30	8.09
S8	27.50	8.60	S20	18.83	3.33
N20	27.17	7.92	S56	18.83	4.22
N55	27.17	5.43	N58	18.67	7.12
S7	27.17	6.54	S39	18.67	8.79
S41	27.17	5.43	N22	18.40	8.06
S17	26.93	5.95	S5	18.40	8.06
S9	26.25	6.18	S35	18.40	5.91
S55	26.25	7.63	N27	17.92	4.85
N25	25.52	7.75	N36	17.92	4.10
N5	25.50	4.47	S23	17.92	4.10
N29	25.50	4.47	S45	17.92	6.60
N39	25.50	4.47	S59	17.50	4.90
N57	25.50	2.58	N24	17.00	6.99
N59	25.50	5.16	S18	17.00	5.96
S19	25.50	3.65	S54	16.25	4.61
S28	25.50	6.32	N30	14.13	6.66
S29	25.50	5.77	S3	13.30	5.73
S31	25.50	4.08	S43	13.30	5.73
S57	25.50	6.17	N23	12.00	4.85
S13	24.71	6.63	N38	8.88	8.88
N49	24.58	5.90	Average	24.5	0.64
N54	24.58	7.84			

**Table 3:** Top five annotated genes nearest/nearby to the most significant SNPs associated with leaf blight incidence rate in Niger. Two top SNPs are statistically significant. Two prevalent bases are listed along with the *p*-value.

Chr	Location	Candidate Gene and Function	Distance (Base Pairs)	Allele	*p*-Value
7	42352720	*Sobic.007G111800* Lysine ketoglutarate reductase trans-splicing related 1 (DUF707)	212,604	Reference: C Alternate: T	0.000000041
5	47989804	*Sobic.005G115200* No annotation Associated PlantFAMs via hmmsearch: Ring finger domain-containing protein	1,282,409	Reference: T Alternate: C	0.000000069
7	50231251	*Sobic.007G116000* Histone-lysine N-methyltransferase SU(VAR)3-9-related Zinc finger	43,037	Reference: A Alternate: G	0.000000096
5	53064984	*Sobic.005G120800* Phosphofructokinase	78,542	Reference: C Alternate: T	0.00000011
10	15300573	*Sobic.010G125000* Steroid nuclear receptor, ligand-binding, putative, expressed	5267	Reference: T Alternate: C	0.00000024

**Table 4:** Top Five Candidate Genes Associated with Leaf Blight Incidence and Severity as Determined by CatBoost. Candidate genes located near the top five SNP markers associated with leaf blight incidence and severity were identified using the CatBoost algorithm in Nigerien and Senegalese sorghum germplasms. The table lists the chromosome (Chr), SNP location, candidate gene name and function, distance between the SNP and gene (in base pairs), reference and alternate alleles, and the CatBoost importance score (%).

Chr	Location	Candidate Gene and Function	Distance (Base Pairs)	Allele	Importance (%)
LB-Incidence					
8	10325034	*Sobic.008G073700* Serine carboxypeptidase 1 precursor	84,236	Reference: C Alternate: G	100
10	6954311	*Sobic.010G081600* Flavonol-3-O-glycoside-7-O-glucosyltransferase 1	5919	Reference: G Alternate: A	49.2
2	27002932	*Sobic.002G144732* LETM1-like	23,252	Reference: A Alternate: G	42.1
1	53951729	*Sobic.001G276700* Oligopeptide transporter	7004	Reference: C Alternate: T	35.6
2	52200082	*Sobic.002G167100* Phosphatidate cytidylyltransferase	34,299	Reference: T Alternate: G	32.7
LB-Severity					
2	47024283	*Sobic.002G155000* Embryo defective 1381	19,113	Reference: A Alternate: G	100
5	38801458	*Sobic.005G112566* Uncharacterized protein	143,175	Reference: A Alternate: C	98.4
9	24964148	*Sobic.009G094600* Uncharacterized protein	22,691	Reference: C Alternate: G	97.5
6	53021705	*Sobic.006G174600* Cis-zeatin O-beta-D-glucosyltransferase	94	Reference: G Alternate: A	94.6
2	12376308	*Sobic.002G104500* Leucine-rich repeat (LRR) protein	610	Reference: T Alternate: C	90.8

**Table 5:** Weather parameters for the two experimental sites.

	Region	
	Dosso	Maradi
Annual rainfall	Average of 700 mm in this region, but up 814 mm in Gaya from March to October (86% between June and September)	550 mm from April to October (66% in July and August)
Climate	Northern Dosso has Sahelian climate while the southern part (Gaya) belongs to the Sahelo-soudanian climate	Sahelian
Mean temperatures during the rainy season	Temperatures (max: 33 °C; min: 24 °C)	Temperatures (max: 28 °C; min: 23 °C)
Soil type	Ferruginous tropical in the most part of this region, but hydromorphous at Bengou and less evoluted at Tara locality	Ferruginous tropical

## Data Availability

The data presented in this study are available upon reasonable request from the authors L.K.P. and C.W.M. The underlying SNP datasets for this sorghum population are publicly available at the Texas Data Repository (TDR) under a CC0 agreement: https://doi.org/10.18738/T8/RGPPGA (accessed on 30 March 2026). The raw sequence data for the C2 accessions are also available in the Sequence Read Archive (SRA) under accession number PRJNA1161677. Supplementary Tables S1 and S2 are provided with the manuscript, and additional processed analysis outputs used in the present study are available from the corresponding authors upon reasonable request.

## Supplementary Materials

The following supporting information can be downloaded at https://www.mdpi.com/article/10.3390/pathogens15040389/s1, Figure S1: Cross-validation and cumulative feature importance for LB-Incidence and LB-Severity; Table S1: Copy of S1_top_80_persent_comlmp_positions_catboost_inc; Table S2: Copy of S2_top_80_persen_cumImp_positions_catboost_sev.
