# Supplementary figures and images for: Genomic Analysis of Resistance to Exserohilum turcicum in Nigerien and Senegalese Sorghum Using GWAS and Machine Learning
Source: Pathogens. 2026 Apr 5;15(4):389. doi: 10.3390/pathogens15040389 (PMC13118907; doi:10.3390/pathogens15040389)

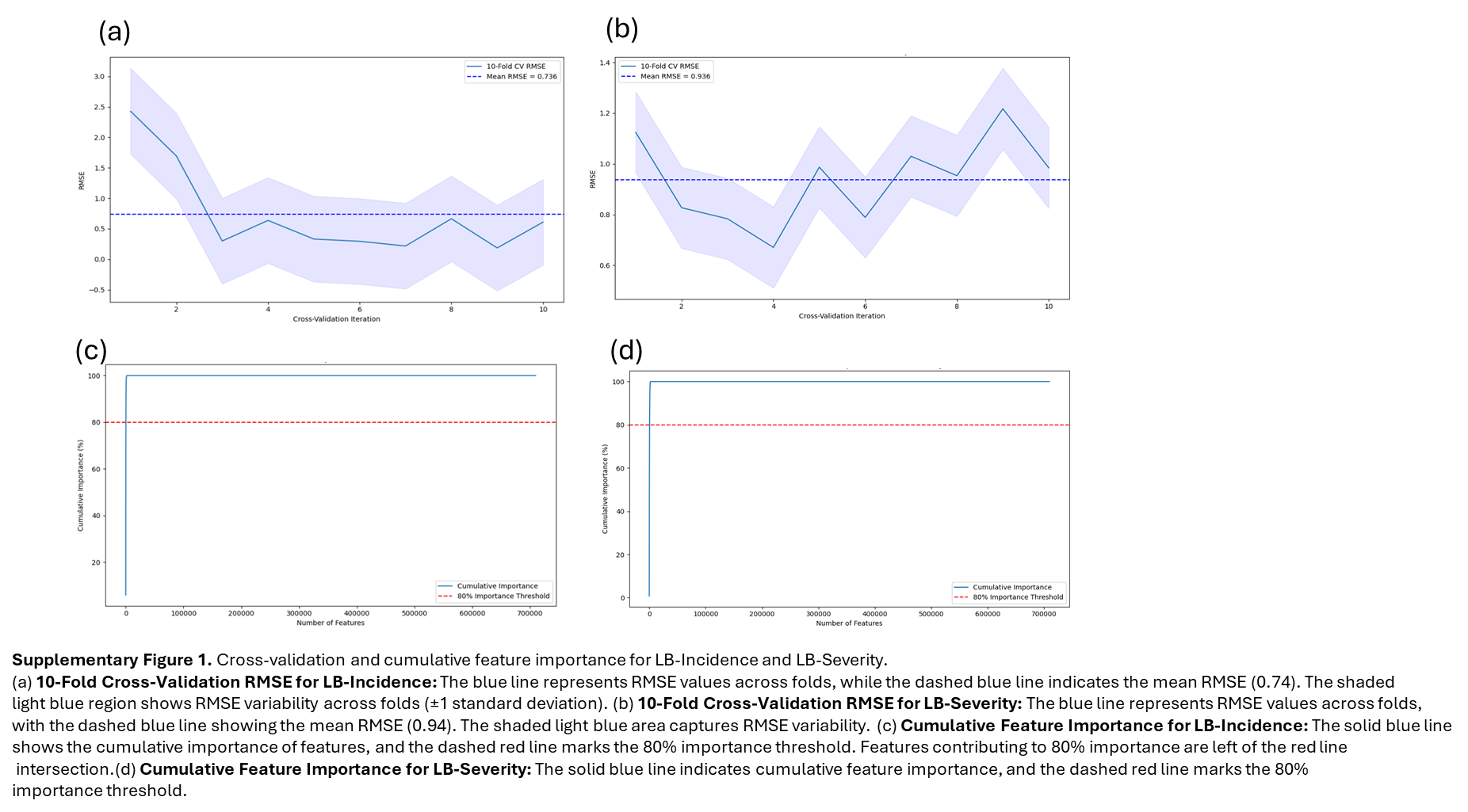

Supplement: Supplementary file 1 [file pathogens-15-00389-s001.zip › pathogens-4195952-supplementary/Supplementary Figure S1.png]
